# Blockade of Platelet-Derived Growth Factor Signaling Inhibits Choroidal Neovascularization and Subretinal Fibrosis in Mice

**DOI:** 10.3390/jcm9072242

**Published:** 2020-07-15

**Authors:** Ye Liu, Kousuke Noda, Miyuki Murata, Di Wu, Atsuhiro Kanda, Susumu Ishida

**Affiliations:** Laboratory of Ocular Cell Biology and Visual Science, Department of Ophthalmology, Faculty of Medicine and Graduate School of Medicine, Hokkaido University, Sapporo 060-8638, Japan; doctorliuye@zju.edu.cn (Y.L.); miyukin@med.hokudai.ac.jp (M.M.); cmueyewd@gmail.com (D.W.); kanda@med.hokudai.ac.jp (A.K.); ishidasu@med.hokudai.ac.jp (S.I.)

**Keywords:** age-related macular degeneration, subretinal fibrosis, platelet derived growth factor receptor-β, choroidal neovascularization, pericyte

## Abstract

Neovascular age related macular degeneration (nAMD) leads to severe vision loss worldwide and is characterized by the formation of choroidal neovascularization (CNV) and fibrosis. In the current study, we aimed to investigate the effect of blockade for platelet derived growth factor receptor-β (PDGFR-β) on the formation of choroidal neovascularization and fibrosis in the laser-induced CNV model in mice. Firstly, the presence of PDGFR-β in CNV lesions were confirmed. Intravitreal injection of PDGFR-β neutralizing antibody significantly reduced the size of CNV and subretinal fibrosis. Additionally, subretinal hyperreflective material (SHRM), a landmark feature on OCT as a risk factor for subretinal fibrosis formation in nAMD patients was also suppressed by PDGFR-β blockade. Furthermore, pericytes were abundantly recruited to the CNV lesions during CNV formation, however, blockade of PDGFR-β significantly reduced pericyte recruitment. In addition, PDGF-BB stimulation increased the migration of the rat retinal pericyte cell line, R-rPCT1, which was abrogated by the neutralization of PDGFR-β. These results indicate that blockade of PDGFR-β attenuates laser-induced CNV and fibrosis through the inhibition of pericyte migration.

## 1. Introduction

Age-related macular degeneration (AMD) is the leading cause of irreversible blindness worldwide in developed countries [1]. The neovascular type of AMD (nAMD) leads to severe vision loss and is characterized by the formation of choroidal neovascularization (CNV), which extends from the choroid into the subretinal space and disrupts Bruch’s membrane and the retinal pigment epithelium (RPE). The formation of CNV is subsequently followed by the development of subretinal fibrosis, resulting in permanent photoreceptor damage and severe vision loss [2,3,4,5]. Among several molecular players participating in the molecular mechanism of CNV formation, vascular endothelial growth factor (VEGF)-A was revealed to play several predominant roles in pathological angiogenesis in nAMD [6]. To date, anti-VEGF therapy has become the first-line treatment for improving visual acuity in patients with nAMD, despite recent studies have elucidated that 10 to 30% of patients with nAMD were non-responders for anti-VEGF drugs [7,8]. Furthermore, a large number of patients receiving anti-VEGF treatment suffered from poor visual prognosis due to subretinal fibrosis [2]. Therefore, alternative therapeutic approaches, which potentially attenuate both CNV and subretinal fibrosis development, would fulfill an unmet medical demand in the treatment of nAMD.

The platelet-derived growth factor (PDGF) signaling system consists of four ligands (PDGF-A, B, C, and D) and two receptors (PDGFR-α and PDGFR-β) [9]. Of these ligands, PDGF-A and PDGF-B form three dimeric isoforms as PDGF-AA, PDGF-AB, and PDGF-BB. PDGFR-α binds all three PDGF isoforms, whereas the PDGFR-β preferentially binds PDGF-BB [9,10]. Of the two cognate receptors that evoke mitogenic signals, PDGFR-β is known to be a more pivotal transducer of cell motility [9,10]. PDGFR-β is expressed on the surface of pericytes and regulates the recruitment, maturation, and survival of pericytes, which share a common basement membrane with endothelial cells to which they provide various growth factors [11,12]. Pericytes not only protect newly formed vessels by stabilization of endothelial cells via paracrine production of growth factors, but also lead to fibrosis formation under pathological conditions in several organs [13,14,15,16,17]. Thus the inhibition of pericytes by blockade of PDGFR-β signaling has been regarded as a novel strategy for fibrogenic diseases such as kidney fibrosis [14].

Pericytes have recently been revealed to function as the primary fibrosis-forming cells in various organs, including the kidney [14,17], lung [13], and spinal cord [15]. Since PDGFR-β signaling is involved in pericyte activation, proliferation, and migration, blockade of pericyte activation with PDGFR inhibitor attenuated interstitial kidney fibrosis in mice [14]. Additionally, it has been reported that pericytes expressing PDGFR-β infiltrate into the subretinal space after CNV induction and subsequently contribute to subretinal fibrosis formation in mice [18]. Interestingly, levels of PDGFs were reported to positively correlate with fibrosis in patients with proliferative diabetic retinopathy (PDR) [19]. Therefore, multiple lines of evidence indicate the potential of PDGF/PDGFR signaling in pericytes as a therapeutic target in ocular angiogenic disorders marked by tissue fibrosis. 

In the current study, we investigated whether blockade of PDGFR-β signaling suppresses the formation of CNV and subretinal fibrosis in laser-induced CNV mice. 

## 2. Materials and Methods

### 2.1. Animals

C57BL/6J mice aged 6–8 weeks from CLEA Japan (Tokyo, Japan) were maintained in the animal facility of Hokkaido University. All animal experiments were conducted in accordance with the guidelines of the Association for Research in Vision and Ophthalmology (ARVO) Statement for the Use of Animals in Ophthalmic and Vision Research. The experimental protocols were approved by the Ethics Review Committee for Animal Experimentation of Hokkaido University (#15-0165).

### 2.2. Laser-Induced CNV Model and Drug Administration

The laser-induced CNV model was generated as described previously [20]. In brief, laser injury (532 nm, 180 mW, 75 μm, 100 ms, Novus Spectra; Lumenis, Tokyo, Japan) was conducted around the optic disc using a slit-lamp delivery system. Four laser spots per eye for quantification of CNV and choroidal fibrosis were performed. The formation of a subretinal bubble at the time of laser injury indicated the rupture of Bruch’s membrane. Immediately after laser injury, 1 μg of PDGFR-β neutralizing antibody (APB5, Thermo Fisher Scientific, Waltham, MA, USA) or isotype-matched IgG (Thermo Fisher Scientific) in 1 μL, was injected into the vitreous cavity of mice. An additional intravitreal injection was given to mice on day 7 for the quantification of subretinal fibrosis formation at 21 days after CNV induction.

### 2.3. Immunofluorescence Microscopy

Seven days after laser injury, eyes were enucleated and fixed in 4% para-formaldehyde for 1 h on ice, incubated in an increasing concentration of PBS/sucrose (5, 10, and 20%), and embedded in frozen section compound (Leica, Exton, PA, USA). After blocking, the sections were incubated with rat anti-PDGFR-β (1:100, Thermo Fisher Scientific) and rabbit anti-NG2 antibody (1:100, Millipore, USA) and subsequently Alexa Fluor 546 secondary antibodies (1:200, Molecular Probes, Waltham, MA, USA). The specimens were mounted in mounting medium, including DAPI (1:500, Vector Laboratories, Burlingame, CA, USA), and examined under a fluorescence microscope (BIOREVO, Keyence, Tokyo, Japan). The number of nucleus surrounded by NG-2 positive signals, corresponding to pericytes, are counted unbiasedly in five consecutive tissue sections, as previously described [17,18].

### 2.4. Quantification of CNV and Subretinal Fibrosis 

The size of CNVs and subretinal fibrosis was measured at 7 and 21 days after laser injury, as described previously [20]. In brief, the anterior segment and retina were removed from the eye after fixation in 4% paraformaldehyde. Four radial incisions were made on the remaining eyecups. The RPE-choroid flatmounts were then incubated with isolectin B4-Alexa488 (1:100, Thermo Fisher Scientific) and rabbit anti-collagen type I antibody (1:100, Rockland Immunochemicals Inc., Limerick, PA, USA) to detect CNV and choroidal fibrosis, respectively. The flatmounts were examined by fluorescence microscope (BIOREVO), and the size of CNV and fibrosis was quantified with microscopy software (BZ-II analyzer, Keyence, Tokyo, Japan).

### 2.5. Quantification of Subretinal Hyper-Reflective Material (SHRM) by Spectral Domain-Optical Coherence Tomography (SD-OCT)

SD-OCT system (Spectralis, Heidelberg Engineering, Heidelberg, Germany) was used for the quantification of SHRM at 7 and 21 days after laser injury. SHRM was defined as the area from the inner border of CNV to the inner border of the RPE [21]. B-scan images containing the maximum size of SHRM were used, and the size of SHRM was measured unbiasedly three times in each CNV spot by two examiners (KN and YL) using ImageJ software (National Institutes of Health (NIH), Bethesda, MD, USA) in a masked fashion. The average size of SHRM in the images was used for analysis.

### 2.6. Cell Migration Assay

Rat retinal pericyte cell line, TR-rPCT1, from transgenic rats harboring the temperature-sensitive SV40 large T-antigen gene, was provided by Fact Inc. (Sendai, Japan) [22]. Pericyte migration was evaluated with an Oris 96-well collagen coated cell migration assay kit (Platypus Technologies, Madison, WI, USA), according to the manufacturer’s protocol. Briefly, cells were seeded in each well and cultured in low glucose DMEM (Fujifilm Wako Pure Chemicals, Tokyo, Japan) supplemented with 10% fetal bovine serum (FBS) at 33 °C and 5% CO_2_. Cells were then starved with DMEM low glucose medium supplemented with 0.5% FBS, for 24 h. After pretreatment with APB5 antibody (0.1 μg/mL, 1 μg/mL and 10 μg/mL) or 10 μg/mL IgG control for 30 min, cells were then treated with PDGF-BB recombinant protein (R&D Systems, Minneapolis, MN, USA) with 10 μg/mL aphidicolin (Fuji Film Wako Pure Chemical) to inhibit cell division. The stoppers were removed to allow cells to migrate into the detection zone. Cells were then incubated for 24 h and stained with PBS containing calcein AM (Dojindo, Kumamoto, Japan) for 1 h. Micrographs were taken with a microscope system (Biorevo). The area of the stained cells that had migrated into the detection zone was measured.

### 2.7. Statistical Analyses

All the results are expressed as the mean ± SEM. Student’s *t*-test was used for statistical comparison between groups, while one-way ANOVA, followed by the Tukey–Kramer method as a post-hoc test, was used for multiple comparison procedures. Differences between means were considered statistically significant when *p* values were <0.05.

## 3. Results

### 3.1. Suppression of CNV Formation by PDGFR-β Blockade

**Figure 1:**
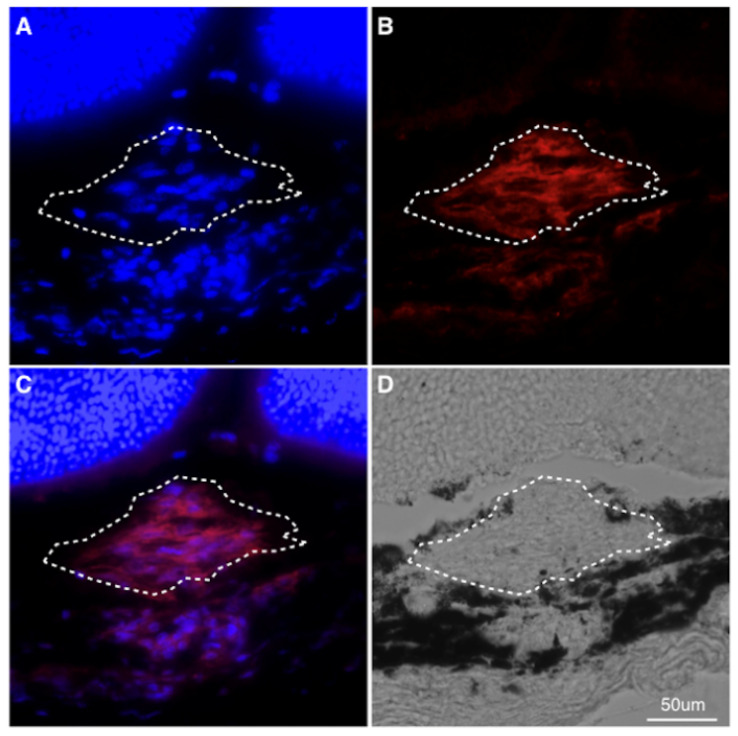
Localization of PDGFR-β in CNV lesions (**A**–**C**) Representative staining of DAPI (blue), PDGFR-β (red) of CNV sections at post-laser day 7. The white dotted line indicates the outline of CNV lesion. (**D**) Phase-contrast image. Scale bar: 50 μm.

**Figure 2:**
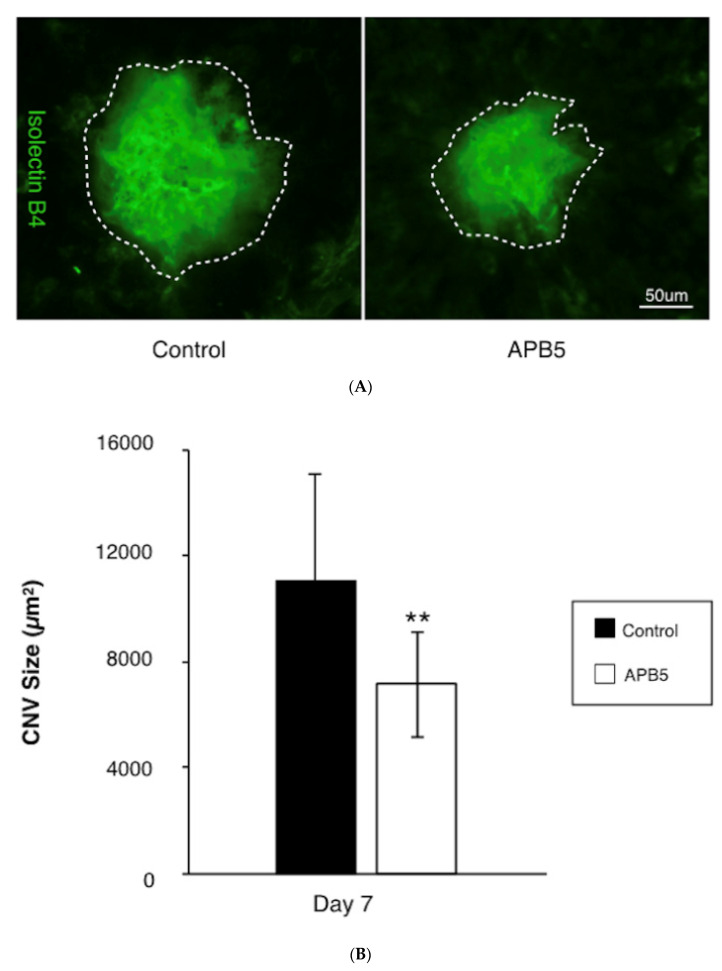
Suppression of CNV formation by PDGFR-β blockade (**A**) Representative micrographs of CNV lesions (isolectin B4, green) in the RPE-choroid flat mounts at post-laser day 7 from mice treated with IgG control or APB5 antibody, respectively. The white dotted line indicates the outline of CNV lesion. Scale bar: 50 μm. (**B**) Quantification analysis of the size of CNV. (** *p* < 0.01, *n* = 12 eyes).

To determine whether PDGFR-β blockade suppresses CNV formation, mice were treated with either APB5, a PDGFR-β neutralizing antibody, or isotype matched IgG immediately after laser injury. Firstly, we confirmed the presence of PDGFR-β in a CNV lesion (Figure 1), and found that intravitreal injections of the APB5 antibody significantly suppressed the formation of CNV at 7 days after laser injury (7154 ± 1859 µm^2^), when compared with isotype-matched IgG-treated controls (11,078 ± 3714 µm^2^, *p* < 0.01, *n* = 12 eyes, Figure 2A,B). This indicates that PDGFR-β blockade suppresses pathological angiogenesis in the laser-induced CNV model, which is consistent with previous reports [23,24].

### 3.2. Attenuation of Subretinal Fibrosis Formation by PDGFR-β Blockade

To examine the effect of APB5 treatment on subretinal fibrosis, we evaluated the temporal changes of subretinal fibrosis using both in vivo imaging in the SD-OCT system and flatmount staining with anti-type I collagen antibody, a marker for fibrotic components. 

**Figure 3:**
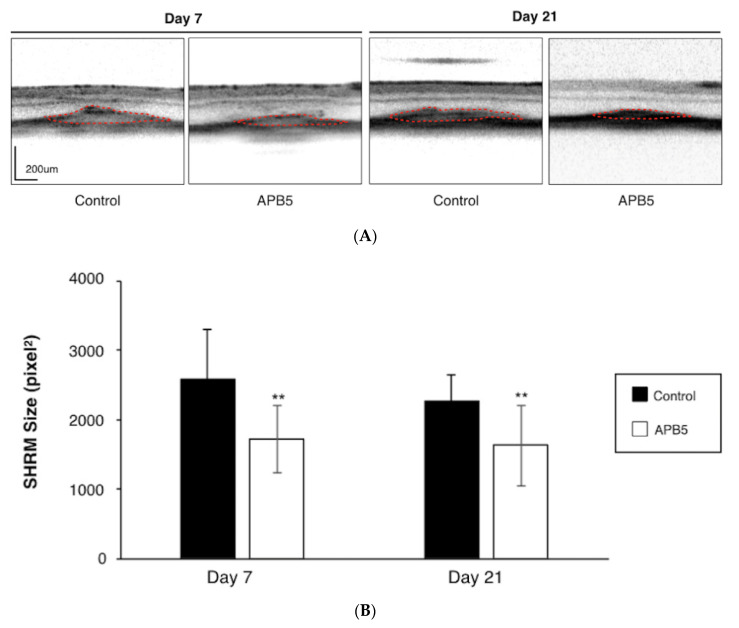
Attenuation of SHRM by PDGFR-β blockade (**A**) Representative OCT images of SHRM at post-laser day 7 and day 21 from mice treated with IgG control or APB5 antibody, respectively. Red dotted line indicates the outline of SHRM. Scale bar: 200 μm. (**B**) Quantification analysis of the size of SHRM. (** *p* < 0.01, *n* = 12 eyes).

SHRM is a morphological feature that displays as hyper-reflective material located between the retina and RPE on OCT, the most widely used device for AMD diagnosis in clinical practice [21]. Using OCT, we evaluated the effect of PDGFR-β blockade on the size of SHRM after laser injury. The average size of SHRM was significantly suppressed in APB5-treated mice (1717.21 ± 390.22 pixels) when compared with IgG-treated controls at 7 days after laser injury (2580.94 ± 716.92 pixels, *p* < 0.01, *n* = 12 eyes, Figure 3A,B). Similarly, at 21 days after laser injury, the average size of SHRM was also significantly suppressed in the APB5-treated group (1626.89 ± 583.29 pixels), compared to that of the IgG-treated control (2259.35 ± 484.87 pixels, *p* < 0.01, *n* = 12 eyes, Figure 3A,B). This data shows that PDGFR-β blockade suppresses the formation of SHRM, a landmark morphological feature in nAMD.

**Figure 4:**
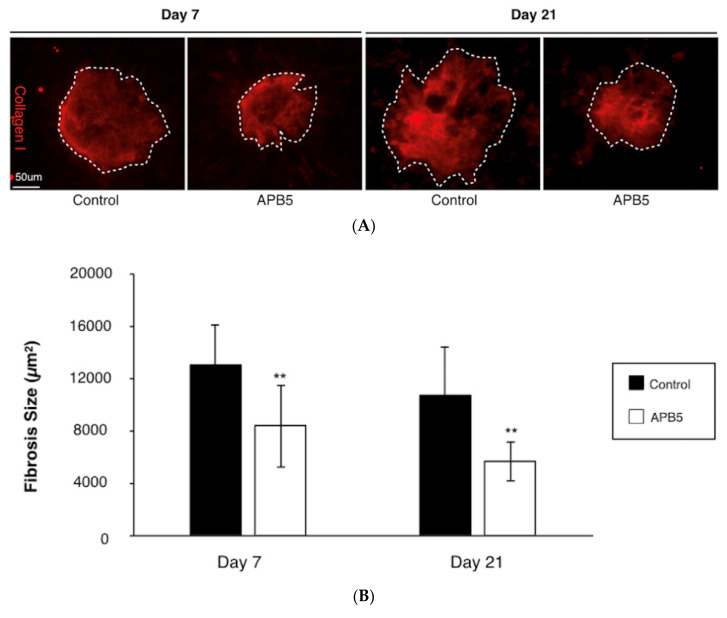
Suppression of subretinal fibrosis by PDGFR-β blockade (**A**) Representative micrographs of subretinal fibrosis lesions (Collagen I, red) in the RPE-choroid flatmounts at day 7 and day 21 post-laser injury, from mice treated with IgG control or APB5 antibody, respectively. The white dotted line indicates the outline of CNV lesion. Scale bar: 50 μm. (**B**) Quantification analysis of the size of subretinal fibrosis at day 7 and day 21 post-laser injury. (** *p* < 0.01, *n* = 12 eyes each).

In support with the SD-OCT evaluation, the average size of subretinal fibrosis was significantly suppressed in APB5-treated CNV mice (8332 ± 2241 µm^2^) when compared with the control group (13,034 ± 3093 µm^2^, *p* < 0.01, *n* = 12 eyes) at 7 days after laser injury (Figure 4A,B). To further assess the impact of APB5 treatment, we measured the size of subretinal fibrosis at 21 days after laser injury. In accordance with the result observed at 7 days after laser injury, the administration of APB5 also suppressed the average size of subretinal fibrosis at 21 days after laser injury; compared to the control group (APB5-treated, 5655 ± 1472 μm^2^ vs. isotype IgG-treated, 10,717 ± 3629 μm^2^, *p* < 0.01, *n* = 12 eyes, Figure 4A,B). The current data demonstrates that PDGFR-β blockade attenuates the formation of subretinal fibrosis in laser-induced CNV mice.

### 3.3. Prevention of Pericyte Recruitment by PDGFR-β Blockade 

**Figure 5:**
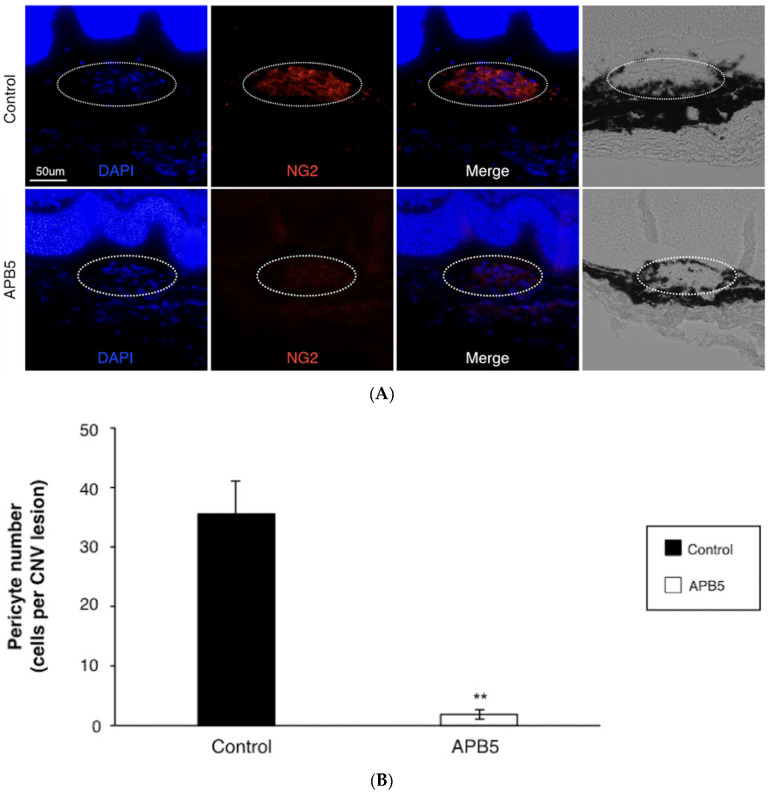
Inhibition of pericyte recruitment by PDGFR-β blockade (**A**) Representative staining of DAPI (blue), NG2 (red) of CNV sections at day 7 post-laser injury from mice treated with IgG control or APB5 antibody, respectively. The white dotted line indicates the outline of CNV lesion. Scale bar: 50 μm. (**B**) Quantification analysis of the recruited pericytes in CNV lesions (** *p* < 0.01, *n* = 8 eyes).

To investigate the mechanism by which PDGFR-β blockade inhibits the CNV formation and subretinal fibrosis, we counted the number of pericytes that infiltrated into the CNV lesion and contributed to subretinal fibrosis formation. NG2 was used as a pericyte marker, as previously described [25,26]. Seven days after laser injury, pericytes were abundantly recruited to the CNV lesions in control CNV mice (36.2 ± 6.5 cells per CNV lesion, Figure 5A,B). However, blockade of PDGFR-β significantly reduced the number of pericytes recruited to the CNV (1.4 ± 0.4 cells per CNV lesion), when compared to the control group (*n* = 8 eyes, *p* < 0.01, Figure 5A,B).

### 3.4. Suppression of Pericyte Migration by PDGFR-β Blockade In Vitro

**Figure 6:**
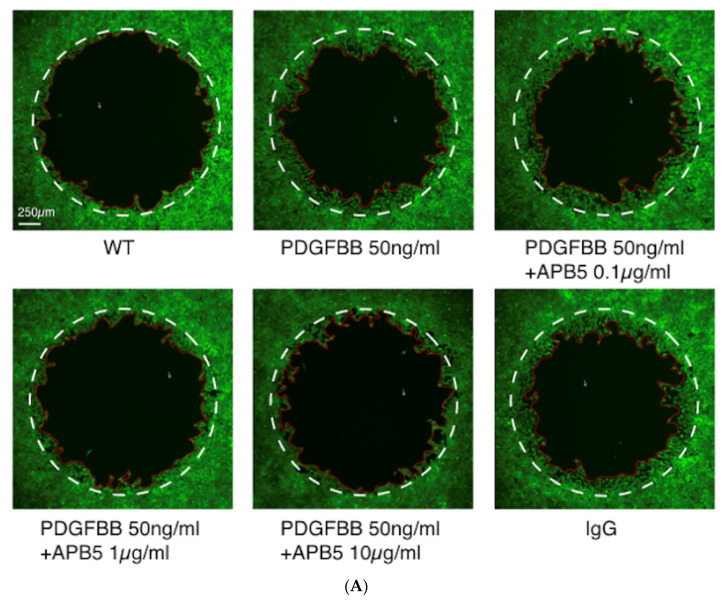

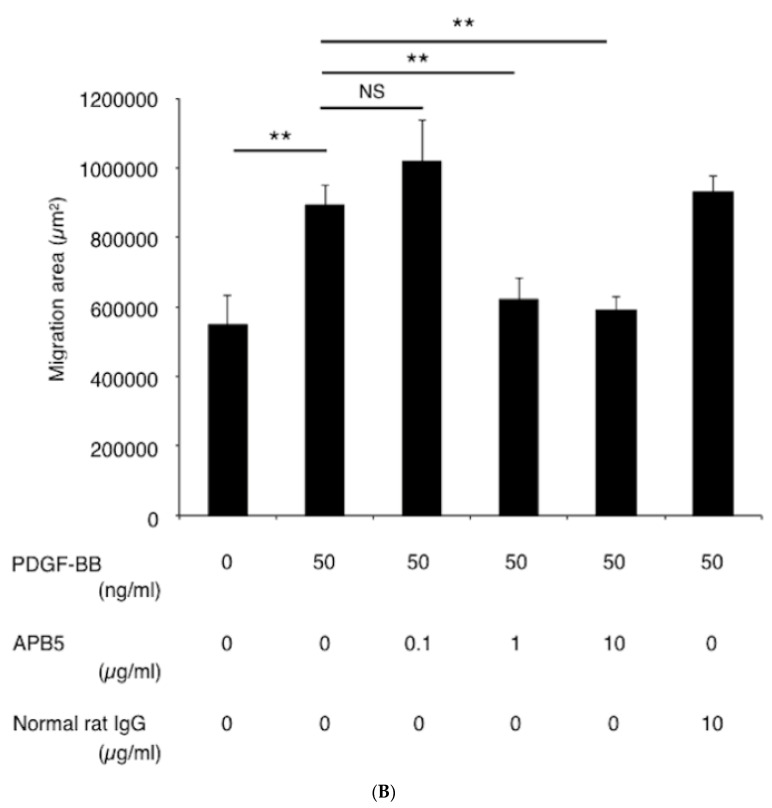
Impact of PDGFR-β blockade on TR-rPCT cell migration stimulated by PDGF-BB. (**A**) Representative micrographs of TR-rPCT cell migration stimulated with PDGF-BB with or without APB5 antibody (0.1 to 10 μg/mL). The white dotted line indicates the baseline, and the red solid line indicates the edge of the migrating cells. Scale bar: 250 μm. (**B**) Cell migration assay in TR-rPCT cell stimulated with PDGF-BB with or without APB5 antibody (0.1 to 10 μg/mL, ** *p* < 0.01, *n* = 4).

To validate the in vivo effect of PDGFR-β blockade on the recruitment of pericytes, we performed in vitro experiments using rat retinal pericyte (TR-rPCT) cells treated with or without PDGF-BB to trigger cell migration. Administration of PDGF-BB significantly enhanced the migration of TR-rPCT1 cells, while blockade of PDGFR-β using APB5 antibody ameliorated the PDGF-BB-induced migration in a dose-dependent manner (Figure 6A,B).

## 4. Discussion

The current study reveals several significant findings concerning the effect of PDGFR-β blockade in CNV and subretinal fibrosis. Blockade of PDGFR-β by intravitreal injection of the PDGFR-β-neutralizing antibody significantly suppressed CNV formation, subretinal fibrosis, and SHRM, a morphological feature seen via OCT in patients with nAMD. Mechanistically, the blockade of PDGFR-β significantly prevents the recruitment of pericytes to the CNV lesions. An in vitro study also supported our in vivo findings that PDGFR-β blockade suppressed PDGF-BB-induced migration of pericytes. Taken together, the current study presents the first evidence that inhibition of PDGF-B signaling by PDGFR-β blockade suppresses CNV and subretinal fibrosis via the inhibition of pericyte migration.

Neovascularization is a multi-step pathological process involving the proliferation and migration of endothelial cells and pericytes. Anti-VEGF treatment, the first-line therapy for CNV, mainly targets vascular endothelial cells and improves visual function during the early stages of treatment. However, it was recently reported that after seven years of anti-VEGF treatment, one-third of patients with nAMD had poor vision at 20/200 or worse [27]. Moreover, 10 to 30% of patients have been reported to be non-responders for anti-VEGF drugs in clinical practice [7,8], suggesting a more complex scenario of pathological angiogenesis than initially anticipated. Indeed, recent studies have reported that blockade of PDGF-B signaling suppresses CNV [23] or, at the very least, provides a synergistic effect when combined with anti-VEGF treatment in mice [28]; suggesting the additional role of PDGF-B signaling in ocular angiogenesis. Previous studies revealed that pericytes, which are regulated by PDGF-B signaling and play pivotal roles in angiogenesis, vascular maturation, and stabilization, are another synergistic cell species on top of vascular endothelial cells basically targeted by anti-VEGF therapies [28,29,30,31]. Notably, it was reported that the level of PDGF-B was significantly higher in the plasma of patients with nAMD [32] when compared with healthy controls, as well as patients with diabetic macular edema, indicating the involvement of PDGF-B signaling in the pathogenesis of nAMD. In accordance with these previous findings, the current study demonstrated the impact of pericyte inhibition via PDGF-B signaling blockade on the formation of CNV and fibrosis.

As a non-invasive device for precise analysis of structural changes in the retina, our group has previously employed OCT to observe the retina for evaluation of the therapeutic effect of novel compounds [33]. In the present study, we used OCT to evaluate the effect of PDGFR-β blockade on the size of SHRM after laser injury. In patients with nAMD, SHRM has been identified as a significant risk factor for subretinal fibrosis formation associated with poor visual acuity [3] and is gradually considered as an OCT biomarker of fibrosis [34,35]. We used an index obtained from SHRM as an evaluation parameter for subretinal fibrosis in laser-induced CNV in mice. As a consequence, we found that blockade of PDGFR-β significantly suppressed SHRM formation at different time points after CNV induction. This is consistent with our finding that PDGFR-β blockade ameliorated the formation of subretinal fibrosis stained by type I collagen in the RPE-choroid flatmounts.

Subretinal fibrosis has recently been revealed as a key pathological event associated with poor visual acuity in CNV; basically a form of fibrovascular proliferation consisting of both vascular and fibrous components, the latter of which is likely resistant to anti-VEGF therapy in clinical practice [2,3]. There is still a controversy about the source of myofibroblasts that produce collagen during fibrosis; however, an increasing body of evidence supports the hypothesis that pericytes are one of the sources of myofibroblasts [11,13,14,15,16,17]. In fact, in other organs, including the kidney, lung, and spinal cord, pericytes were found to drive fibrosis and were therefore regarded as a target for inhibition of fibrosis [11,13,14,15,16,17]. In the current study, using the pericyte marker NG2, we found abundant expression of NG2-positive pericytes in control mice at 7 days after laser injury, indicating the recruitment of pericytes during the development of CNV and subretinal fibrosis. However, the administration of the PDGF-β-neutralizing antibody significantly prevented the recruitment of pericytes. To further confirm our in vivo finding, we also conducted in vitro migration analysis in rat pericytes under the stimulation of PDGF-BB. Notably, blockade of PDGFR-β suppressed the pericyte migration induced by PDGF-BB in a dose-dependent manner.

Recently, a clinical trial has been conducted to assess the efficacy of anti-PDGF and anti-VEGF combination therapy with anti-VEGF monotherapy in patients with nAMD. As a consequence, no superiority was found in the combination group and precluded the further clinical application of anti-PDGF therapy in nAMD [36]. However, the results of these trails should be interpreted with caution. The trails set the 12-month visual acuity as primary endpoint outcome. The 12-month duration might be sufficient to evaluate the therapeutic effect for neovascularization, but not for fibrotic scar, which occurs in a late stage of nAMD or after receiving anti-VEGF therapy [3]. Therefore, the potential role of PDGF blockade to prevent scar formation in nAMD is warranted to be evaluated in future studies.

The present study has several limitations. First, as the current study was conducted in mice, it is likely that the SHRM observed in the current study is not identical to that seen in patients with nAMD, which is likely composed of the elements including fluid, fibrin, blood, scar, and CNV [21]. Second, whereas we found that PDGFR-β signaling plays a pivotal role in the recruitment of pericytes during subretinal fibrosis formation, the detailed molecular mechanisms underlying this cascade remains elusive. Further analysis is necessary to elucidate the role of PDGFR-β signaling in subretinal fibrosis formation.

In conclusion, blockade of PDGFR-β signaling attenuates laser-induced neovascularization and subretinal fibrosis via pericyte deactivation. This suggests that, aside from vascular endothelial cells, pericytes might be a promising therapeutic target for the treatment of nAMD in clinical practice.
